# Effects of access to radiology in out-of-hours primary care on patient satisfaction and length of stay

**DOI:** 10.1080/13814788.2021.1959911

**Published:** 2021-08-09

**Authors:** Martijn H. Rutten, Paul H. J. Giesen, Willem J. J. Assendelft, Gert Westert, Marleen Smits

**Affiliations:** a Radboud University Medical Centre, Radboud Institute for Health Sciences, Scientific Centre for Quality of Healthcare (IQ healthcare), Nijmegen, The Netherlands; b Department of Primary and Community Care Nijmegen, Radboud University Medical Centre, Radboud Institute for Health Sciences, The Netherlands

**Keywords:** Radiology, primary care, emergency services, out-of-hours medical care, organisational efficiency

## Abstract

**Background:**

Direct access to hospital radiology facilities by general practitioner (GP) cooperatives is known to decrease the number of emergency department referrals, but the effects on length of stay (LOS; time from patient arrival at GP cooperative till departure to home) and patient experiences are unclear.

**Objectives:**

To provide insight into the LOS and experiences of trauma patients with an indication for radiology at GP cooperatives with and without access to radiology.

**Methods:**

A multi-methods observational study in April 2014–October 2015 at six GP cooperatives in The Netherlands, covering three organisational models for access to radiology: no direct access, limited access and unlimited access. Patient experiences were measured with a questionnaire. Patient records were analysed for background characteristics, radiology outcomes, referral and LOS.

**Results:**

In total 657 patients were included, 232 no direct access model, 307 limited access model and 118 unlimited access model. The mean LOS was 99 minutes, with a significant difference between GP cooperatives without access to radiology (121 minutes), with limited access (86 minutes), and with unlimited access (90 minutes). The differences were larger for patients without radiological abnormalities. On a ten-point scale, patients rated GP cooperatives with unlimited access to radiology higher (8.62) than those without access (8.36) or with limited access (8.39).

**Conclusion:**

Access to radiology by GP cooperatives seems to reduce the length of stay and is slightly more appreciated by patients. GP cooperatives with unlimited access seem to provide the most efficient and best-valued care, contributing to more patient-centred care.

KEY MESSAGES

- Direct access to radiology by the GP cooperative reduces the total LOS of trauma patients who require conventional radiology imaging.
- Patients slightly more appreciate GP cooperatives with unlimited access to radiology than those without or with limited access (in restricted time frames).

## Introduction

There are various organisational models for out-of-hours primary care in Europe, of which the general practitioner (GP) cooperative is predominant [1]. In these large-scale organisations, GPs take turns being on duty during out of hours for the patient populations of all participating GPs (15–250 GPs per cooperative). In The Netherlands, GP cooperatives are increasingly located on the site of hospital emergency departments (EDs), which creates possibilities for collaboration [2,3]. An example is the triage and treatment of self-referring patients by the GP cooperative instead of the ED [3]. Another possibility is direct access to hospital diagnostics, such as radiology. GPs are often consulted for musculoskeletal trauma, in which conventional radiology may help rule out a fracture or luxation [4]. In The Netherlands, during office hours the GP in the general practice has direct access to the radiology facilities of the hospital. Only in case of radiological abnormalities does the patient receive a referral to the ED; otherwise further treatment is offered by the GP in the general practice. However, during out of hours, patients often need to be referred to the ED, as GP cooperatives often do not have access to conventional radiology facilities [5–7]. A lack of access to hospital diagnostics causes unnecessary contacts with EDs, which are already struggling with overcrowding [8–10]. Moreover, the contribution from the patient’s annual deductible is higher and the total length of stay (LOS) is possibly longer [11,12]. Longer waiting times have been associated with lower patient satisfaction [13].

In past years, several GP cooperatives in The Netherlands have gained direct access to the hospital’s radiology facilities, without the need to refer patients to the ED [5]. Mostly, the access is limited to certain time frames [5,6]. Previous studies into access to radiology by GP cooperatives in The Netherlands have shown that GPs used the radiology facilities adequately [6], more patients were maintained under treatment of the GP [6] and the total number of ED contacts was reduced by 4.5% [7]. It might also positively influence patient satisfaction and LOS. These are indicators of patient centredness, one of the six domains of quality of care [14]. This study aimed to examine the effects of different organisational models of radiology access by the GP cooperative on patient experiences and LOS. We hypothesised that GP cooperatives with unlimited access to radiology would have higher patient satisfaction and shorter LOS than GP cooperatives with limited access (in restricted time frames) and without direct access to radiology.

## Methods

### Study design and population

We performed an observational study combining patient registration analysis with a survey among trauma patients referred by the GP cooperative for conventional radiology (X-ray) in April 2014–October 2015. The study was carried out in a convenience sample of six GP cooperatives covering three organisational models: one with unlimited access, three with limited access (in restricted time frames) and two without direct access to radiology. The study period in each GP cooperative varied from 3 to 5 months (Table 1).

**Table 1.** Models of access of GP cooperatives to radiology in The Netherlands, study period and background information.

Access to Radiology		Study period	Background information
None	A	April–July 2014	Located in the eastern part of The Netherlands. No access to conventional radiology by the GP cooperative. Referral to the ED necessary.
	B	October–December 2014	Located in the south-eastern part of The Netherlands. No access to conventional radiology by the GP cooperative. Referral to the ED necessary.
Limited	C	December 2014–April 2015	Located in the south-west of The Netherlands. GP cooperative access to conventional radiology during weekends and public holidays only, possibilities between 11 and 12 a.m. and 5 and 6 p.m. Analysis by hospital radiologist. Outside these hours referral to the ED for conventional radiology is necessary.
	D	May–June 2015	Located in the west of The Netherlands. GP cooperative access to conventional radiology on weekdays between 5 and 8 p.m. and during weekends and public holidays between 10 a.m. and 8 p.m. Analysis by hospital radiologist. Outside these hours referral to the ED for conventional radiology is necessary.
	A′	June–September 2015	Located in the eastern part of The Netherlands. GP cooperative access to conventional radiology on weekdays and during weekends and public holidays, with nightly exclusion. Analysis by a radiologist in an associated hospital elsewhere. Outside these hours referral to the ED for conventional radiology is necessary.
Unlimited	E	July–October 2015	Located in eastern part of The Netherlands. Unlimited access by the GP cooperative during their opening hours. Analysis by the hospital radiologist.

Note: A and A′ are the same organisation.

### Selection of study subjects

In all three models, all patients with trauma in which the GP and patient decided to take an X-ray were eligible for inclusion. There were no exclusion criteria. The GP informed the patients about the study and asked them to sign an informed consent form.

### Procedure

After the GP consultation, included patients were directed to the GP cooperative desk, where a study number was assigned to them. This number was written on the informed consent form and patient questionnaire. Patients were requested to return the form and questionnaire at the GP cooperative desk directly after their treatment. The desk assistant listed the time of emission and return of the questionnaire on the questionnaire form. The informed consent form, the (anonymous) patient questionnaire and study number list with personal data were stored separately by the desk assistant. Forms returned outside the GP cooperative opening hours could be dropped in a closed post box or returned by post addressed to the GP cooperative.

### Measurements

The patient questionnaire measured patient experiences with the provided care using a 4-point scale of agreement (1 = not at all; 2 = a little; 3 = largely; 4 = totally) or ten-point scale of quality (1 = very bad; 10 = excellent). The questionnaire was based on the Consumer Quality Index (CQI) [15]. Two questions about the experienced collaboration between GP cooperative and ED were added and filled in only by patients referred to the ED. The questionnaire consisted of 21 questions, which could be answered in less than 5 minutes.

To examine LOS we used the time of start of the consultation at the GP cooperative as registered in the patient record as starting point (A). The questionnaire and informed consent form were handed over to the patient after the GP cooperative consultation (B). The time of return of the questionnaire was used as the endpoint for LOS (C). Questionnaires returned by post or post box (16%) were not used in the LOS analysis.

### Data extraction

Routinely registered data were extracted from the GP cooperatives’ electronic registration systems, including age, gender, affected body part, and referral to the ED. The imaging report of the radiologist was obtained from the hospital administration system. For patients who were referred to the ED, the ED file was obtained as well, to verify the diagnosis.

### Statistical analyses

Descriptive statistics were used to describe the patient population. Patient characteristics, LOS and patient experiences for the three models were compared with T-tests and chi-square tests. To calculate percentages of agreement on the items measuring patient experiences, the four-point answering scale was dichotomised into agree (‘largely’ plus ‘totally’) and not agree (‘not at all’ plus ‘a little’). Testing, however, was performed on the total scale. The LOS and perceived problems with LOS were calculated for the subgroups of patients with and without radiological abnormalities. For all data analyses IBM SPSS 22 (Statistical Package for Social Sciences) was used. *P* values less than 0.05 were considered statistically significant.

### Ethics

The Ethical Research Committee of the Radboud University Medical Centre Nijmegen was consulted and concluded that this study does not fall within the remit of the Dutch Medical Research Involving Human Subjects Act [Wet Mensgebonden Onderzoek] (2014/219].

## Results

### Patient characteristics

**Figure 1.**
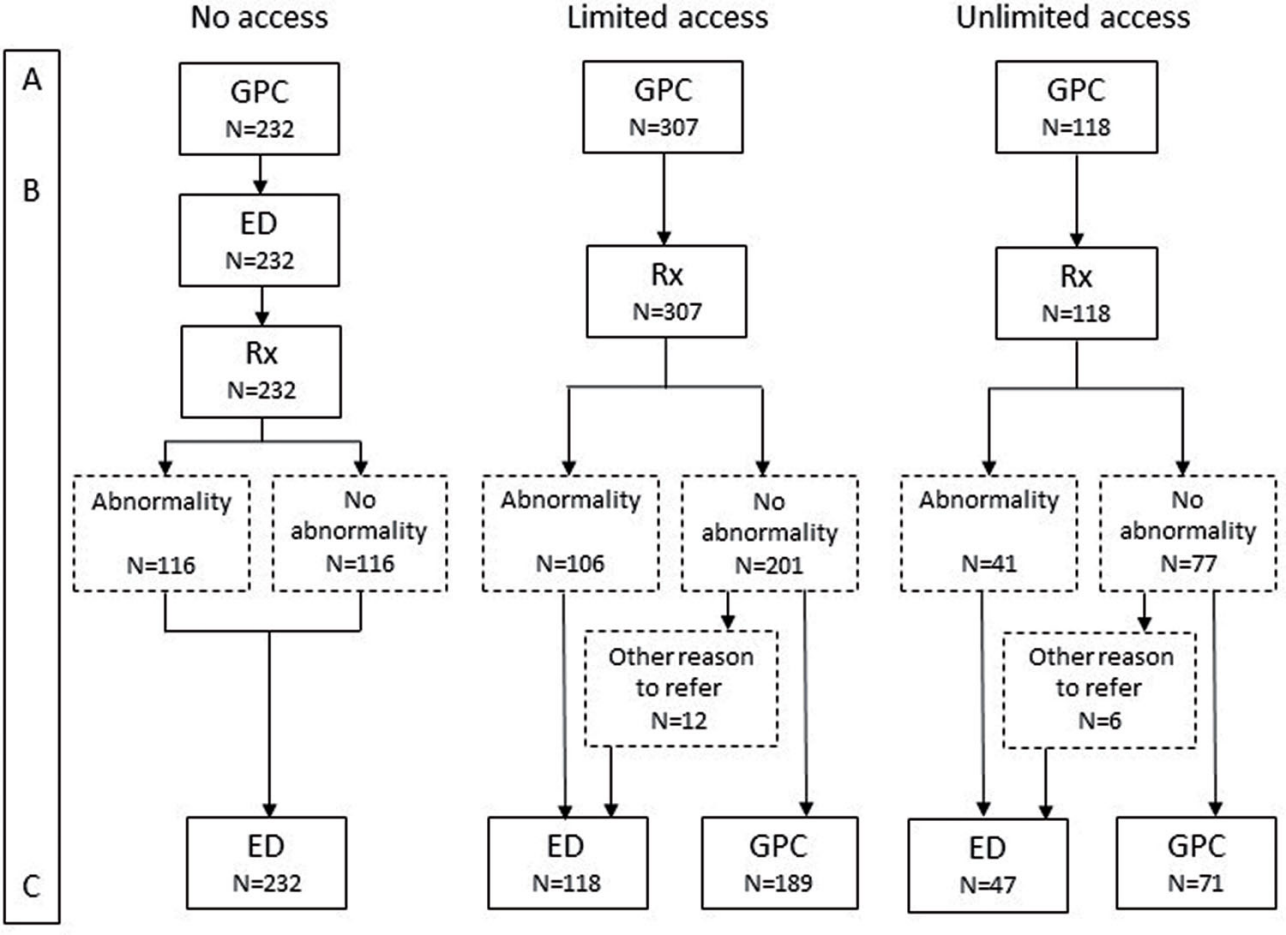
Patient flow chart per organisational model for general practitioner cooperative access to radiology: no access, limited access (in restricted time frames) and unlimited access (all opening hours). (A) Appointment time of the patient at the GPC (start point). (B) Issue of questionnaire and informed consent form to the patient. (C) Return of questionnaire and informed consent form (endpoint).

We included 657 trauma patients within the three organisational varieties for accessibility to conventional radiology (X-ray) by the GP cooperative: 232 patients (35%) in the model without access, 307 patients (47%) in the model with limited access and 118 (18%) in the model with unlimited access (Figure 1). The mean age was 31.3 years and 55.5% were women. Most injuries concerned extremities (91.0%). The population was similar within the three organisational models regarding age and gender, but differed in the percentage of wrist and foot/toe injuries (Table 2).

**Table 2.** Patient characteristics per organisational model with respect to access to radiology by the GP cooperative.

Characteristic	No access *N* = 232	Limited access *N* = 307	Unlimited access *N* = 118	Total *N* = 657
Age mean (SD)	31.3 (22.9)	30.6 (22.2)	33.4 (20.6)	31.3 (22.2)
Gender female *N* (%)	134 (58.3)	170 (55.4)	59 (50.4)	363 (55.5)
Affected body part *N* (%)				
Wrist*	58 (25.2)	63 (20.7)	16 (13.6)	137 (21.0)
Foot/toes***	23 (10.0)	74 (24.3)	35 (29.7)	132 (20.2)
Hand/fingers	41 (17.8)	60 (19.7)	26 (22.0)	127 (19.4)
Ankle	44 (19.1)	34 (11.1)	17 (14.4)	95 (14.5)
Elbow/lower arm	22 (9.6)	23 (7.5)	8 (6.8)	53 (8.1)
Shoulder/clavicle	15 (6.5)	19 (6.2)	9 (7.6)	43 (6.6)
Knee/lower leg	17 (7.4)	17 (5.6)	2 (1.7)	36 (5.5)
Other	10 (4.3)	15 (4.9)	5 (4.2)	30 (4.6)
Radiological abnormality** *N* (%)	116 (51.3)	106 (34.5)	41 (34.7)	263 (40.4)
Referral to ED*** *N* (%)	226 (100)	118 (38.4)	47 (39.8)	391 (60.0)

*P < 0.05; **P < 0.01; ***P < 0.001.

In total, 40.4% of the patients were diagnosed as having a fracture or luxation (*N* = 263). The outcomes differed significantly between the model without access and models with (limited) access. The percentage of radiological abnormalities (fractures and luxations) was 51.3% in the model without access to radiology, 34.5% in the model with limited access and 34.7% in the model with unlimited access. The ED referral rate for GP cooperatives without access to radiology was (logically) 100%, compared to 38.4% in the case of limited access and 39.8% in unlimited access (Table 2).

### Length of stay

The mean total LOS for all included patients was 99 minutes. The total LOS in the model without direct access to radiology (121 minutes) differed significantly from the models with limited access (86 minutes) and unlimited access (90 minutes). The difference between the models in mean LOS was the largest for patients without radiological abnormalities. In the model without direct access these patients had to visit the ED and their mean total LOS was 112 minutes. In the (un)limited access models, patients without radiological abnormalities maintained under treatment of the GP cooperative; their mean total LOS was 70 minutes in the limited access model and 63 minutes in the unlimited access model (Table 3).

**Table 3.** Length of stay (in minutes) and patient experiences within the different organisational models with respect to access to radiology by the GP cooperative.

Outcome	No access	Limited access	Unlimited access	Total
Length of stay				
All patients*** ^a^	*N* = 191	*N* = 259	*N* = 104	*N* = 651
Mean (95%CI)	121 (114–128)	86 (80–92)	90 (79–102)	99 (95–103)
Range	32–300	15–245	17–270	15–300
Patients without radiological abnormality*** ^a^	*N* = 103	*N* = 170	*N* = 66	*N* = 339
Mean (95%CI)	112 (103–121)	70 (64–75)	63 (54–73)	81 (77–86)
Range	32–270	15–235	17–205	15–270
Patients with radiological abnormality	*N* = 88	*N* = 89	*N* = 38	*N* = 215
Mean (95%CI)	132 (121–143)	117 (107–127)	137 (118–157)	127 (120–134)
Range	40–300	30–245	37–270	30–300
Length of stay perceived as a problem	*N* = 232	*N* = 307	*N* = 118	*N* = 657
All patients *N* (%)	113 (52.6)	121 (43.1)	57 (51.4)	291 (47.9)
Patients without radiological abnormality *N* (%)	55 (51.9)	69 (38.1)	33 (45.8)	157 (43.7)
Patients with radiological abnormality *N* (%)	58 (53.2)	52 (52.0)	24 (61.5)	134 (54.0)
Informed about expected length of stay *N* (%)	75 (34.9)	105 (37.8)	31 (28.2)	211 (35.0)
Experiences with GPC professional				
Taken seriously *N* (%)	230 (100)	285 (96.4)	113 (98.2)	651 (98.0)
Confidence in expertise *N* (%)	226 (99.2)	298 (97.7)	115 (100)	650 (98.6)
Sufficient time *N* (%)	226 (99.2)	299 (97.7)	115 (100)	649 (98.6)
Experiences with ED professional				
Taken seriously *N* (%)	209 (89.6)	96 (97.0)	35 (100)	346 (98.3)
Confidence in expertise *N* (%)	210 (99.1)	96 (97.9)	35 (100)	345 (98.8)
Sufficient time *N* (%)	202 (95.8)	93 (93.9)	34 (97.1)	345 (95.4)
Grade GPC*^b^				
Mean (95%CI)	8.36 (8.22–8.50)	8.39 (8.27–8.50)	8.62 (8.43–8.81)	8.42 (8.34–8.50)
Range	5–10	3–10	6–10	3–10
Grade ED				
Mean (95%CI)	8.07 (7.91–8.24)	8.22 (7.94–8.49)	8.03 (7.55–8.51)	8.11 (7.97–8.24)
Range	3–10	0–10	2–10	0–10
Grade cooperation GPC–ED				
Mean (95%CI)	7.7 (7.37–8.03)	8.02 (7.74–8.30)	8.07 (7.68–8.46)	7.91 (7.72–8.10)
Range	0–10	0–10	5–10	0–10

*P < 0.05; **P < 0.01; ***P < 0.001.

^a^Significant difference between model without access and models with (un)limited access.

^b^Significant difference between model without access and model with unlimited access.

GPC: general practitioner cooperative; Rx: conventional radiology; ED: emergency department; LOS: length of stay.

### Patient experiences

About half of the total population (47.9%) considered the LOS as problematic. In the models with (un)limited access, patients without radiological abnormalities considered the LOS less often as a problem (38.1% limited access model; 45.8% unlimited access model) than patients who had to visit the ED because of radiological abnormalities (52.0% limited access model; 61.5% unlimited access model). In the model without direct access, there was almost no difference in perceived problems with LOS between patients with and without radiological abnormalities (53.2 vs 51.9%) (Table 3).

The mean grade (on a ten-point scale) for care at the GP cooperative was 8.42 (*n* = 644). For patients who visited the ED the mean grade for care was 8.11 (*n* = 343). The mean grade for the collaboration between ED and GP cooperative was 7.91 (*n* = 240, only ED referred patients). The GP cooperative with unlimited access obtained a significantly higher grade (8.62) than GP cooperatives without access (8.36) or with limited access (8.39). Only in the model with unlimited access did none of the respondents give the GP cooperative a grade below 6 (Table 3). We did not find a correlation between LOS and patient satisfaction (data not shown).

Most patients indicated that they felt taken seriously by the professionals (GP cooperative 98% and ED 98.3%) and that they had confidence in the medical expertise (GP cooperative 98.6% and ED 98.8%). According to the patients, professionals at the GP cooperative and ED had sufficient time (GP cooperative 98.6% and ED 95.4%) (Table 3).

## Discussion

### Main findings

Our study shows that access to conventional radiology by the GP cooperative is related to a shorter LOS, particularly for patients without radiological abnormalities. The patients were overall satisfied with the delivered care by the GP cooperative, ED and their collaboration. The GP cooperative with unlimited access to radiology had higher satisfaction ratings than those with limited or without access. Patients felt taken seriously and had confidence in the expertise of the professionals working at the GP cooperative and ED.

### Strengths and limitations

To our knowledge, this study is the first to investigate the effects of access to radiology by the GP cooperative on LOS and patient satisfaction. It supplements a previously published study of the impacts on referrals [6]. The study was performed in 2014–2015. Since then, more GP cooperatives have gained direct access to radiological facilities. However, we do not believe that repeating the study now would provide other results.

We could not perform an experimental study, but investigated the current situation in a convenience sample of six GP cooperatives. The number of GP cooperatives varied from only one to three per model. Due to the study design, we were not able to demonstrate causal relationships. Organisational differences between the GP cooperatives could have influenced the results. Moreover, the sampling method limits the generalisability of the results to other regions and countries.

The total number of selected patients was not recorded, making it impossible to calculate a response rate. Selection bias could have occurred because patients admitted to the hospital possibly did not return their questionnaires and forms. Furthermore, 16% of all patients were lost to analysis in the calculation of the LOS. For these patients we did not know a LOS endpoint because they returned their questionnaire by post (box). The patient characteristics of these patients were similar to the total study population. We do not believe this loss of patients has influenced the results.

Finally, the time to fill out the questionnaire (less than 5 minutes) was included in the LOS because we used the time of return of the questionnaire as the endpoint of care. The LOS is, therefore, somewhat overestimated. However, this applied to all three models equally, so it has not influenced our comparative analyses.

### Interpretation of the study results in relation to existing literature

Thijssen et al. [12] examined the LOS of patients at the ED in The Netherlands and found a mean of 130 minutes per ED visit, which is relatively low compared to other western countries (176–480 minutes). For trauma patients, they reported a mean ED LOS of 91 minutes [12]. In our study, the mean LOS for patients attending both the GP cooperative and ED was 120 minutes in total. The difference in LOS of 29 minutes is understandable because our study included the GP cooperative visit, radiological diagnostic examination and evaluation, ED visit and possible waiting times in between. Despite the relatively short LOS, almost half of the patients in our study considered this problematic.

The patients in our study highly appreciated the care at the GP cooperative and ED in general (mean grades 8.4 and 8.1, respectively). This aligns with previous Dutch patient satisfaction studies at the GP cooperative [16,17] and ED [18]. It has been found that the strongest predictor of ED patient satisfaction is how satisfied the patient is with interpersonal interactions with ED physicians and nurses [13]. Interactions of patients with healthcare providers have not been examined in our study. However, patients reported they felt taken seriously and had high confidence in the expertise of the professionals. This most likely has had positive effects on the relatively high satisfaction grades reported in our study. Waiting time has also been revealed as an important predictor for satisfaction: if these are longer than what patients expect or deem appropriate, dissatisfaction is likely to arise [18]. We did not find any correlation between LOS and patient satisfaction.

### Implications for practice and further research

Crowding of EDs is a problem in many western countries [8–10]. Multiple factors have been associated with ED crowding, of which non-urgent ED visits are frequently mentioned. Some of these non-urgent visits are made by self-referred patients presenting at the ED rather than in primary care [19–22]. Increasingly, GP cooperatives and EDs are co-locating and collaborating, giving opportunities to redirect the self-referred patients from ED to GP cooperative [2]. The GP cooperative can safely treat 76% of the redirected self-referrals [2]. The referral rate could be further reduced by giving the GP cooperative access to radiology, which is standard during daytime. GPs with access to radiology use the diagnostics in a restrained way and cause a reduction in ED referrals [5,6]. A reduction of 4.5% of the total ED attendance has been shown even in limited access to radiology [7].

Access to radiology by the GP cooperative should be considered to provide efficient and patient-centred care and to prevent non-urgent care at the ED. On the other hand, initiatives to redirect low-complex care from the ED to primary care settings increase workload. It is advisable to invest in primary care setting to keep this redirection sustainable.

## Conclusion

This study shows that access to conventional radiology (X-ray) by the GP cooperative is related to a lower mean LOS and is slightly more highly valued by patients. GP cooperatives with unlimited access seem to provide the most efficient and best-valued care, contributing to patient-centred care.
